# Adenosine stress CMR with spiral pulse sequences accurately detect CAD

**DOI:** 10.1186/1532-429X-14-S1-P13

**Published:** 2012-02-01

**Authors:** Michael Salerno, Christopher Sica, Craig H Meyer, Christopher M Kramer

**Affiliations:** 1 Cardiovascular Medicine, University of Virginia, Charlottesville, VA, USA; 2 Radiology, University of Virginia, Charlottesville, VA, USA; 3 Center for NMR Research, Penn State Hershey School of Medicine, Hershey, PA, USA; 4 Biomedical Engineering, University of Virginia, Charlottesville, VA, USA

## Summary

We demonstrate that adenosine stress CMR using variable density spiral perfusion pulse sequences accurately detects obstructive coronary artery disease. These pulse sequences produce high quality perfusion images with minimal artifacts resulting in high diagnostic accuracy.

## Background

Adenosine stress perfusion imaging with CMR has been limited by motion-induced dark-rim artifacts, which may be mistaken for true perfusion abnormalities. We have previously demonstrated that spiral pulse sequences can produce high quality first-pass perfusion images.[1] We have further improved this spiral technique using high-resolution variable density spiral trajectories and a novel density compensation function which reduces Gibbs ringing.[2] We aimed to test the clinical performance of this improved spiral perfusion pulse sequences with adenosine stress for the detection of obstructive coronary artery disease (CAD).

## Methods

CMR perfusion imaging was performed during adenosine stress (140µg/kg-min) and at rest on a Siemens 1.5T Avanto scanner in 23 subjects scheduled to undergo cardiac catheterization for evaluation of chest pain. Subjects with prior coronary artery bypass surgery were excluded. Perfusion images were acquired during injection of 0.1mmol/kg Gd-DTPA at 3 short-axis locations using a saturation recovery (SR) interleaved variable-density spiral pulse sequence with an integrated field-map for off-resonance correction during reconstruction. Sequence parameters included: SR time 80 ms, FOV 320-340mm^2^, nominal resolution 2.0 mm^2^, 8 spiral interleaves, FA 30^0^, TR/TE 10ms/1ms. Cine and late gadolinium enhanced (LGE) images were also obtained using standard methodology. All subjects underwent cardiac catheterization following the CMR and significant stenosis was defined as >50%. Two blinded reviewers evaluated the spiral perfusion images for the presence of adenosine-induced perfusion abnormalities. Image quality was graded on a 5 point scale (1 - poor to 5- excellent).

## Results

**Figure 1:**
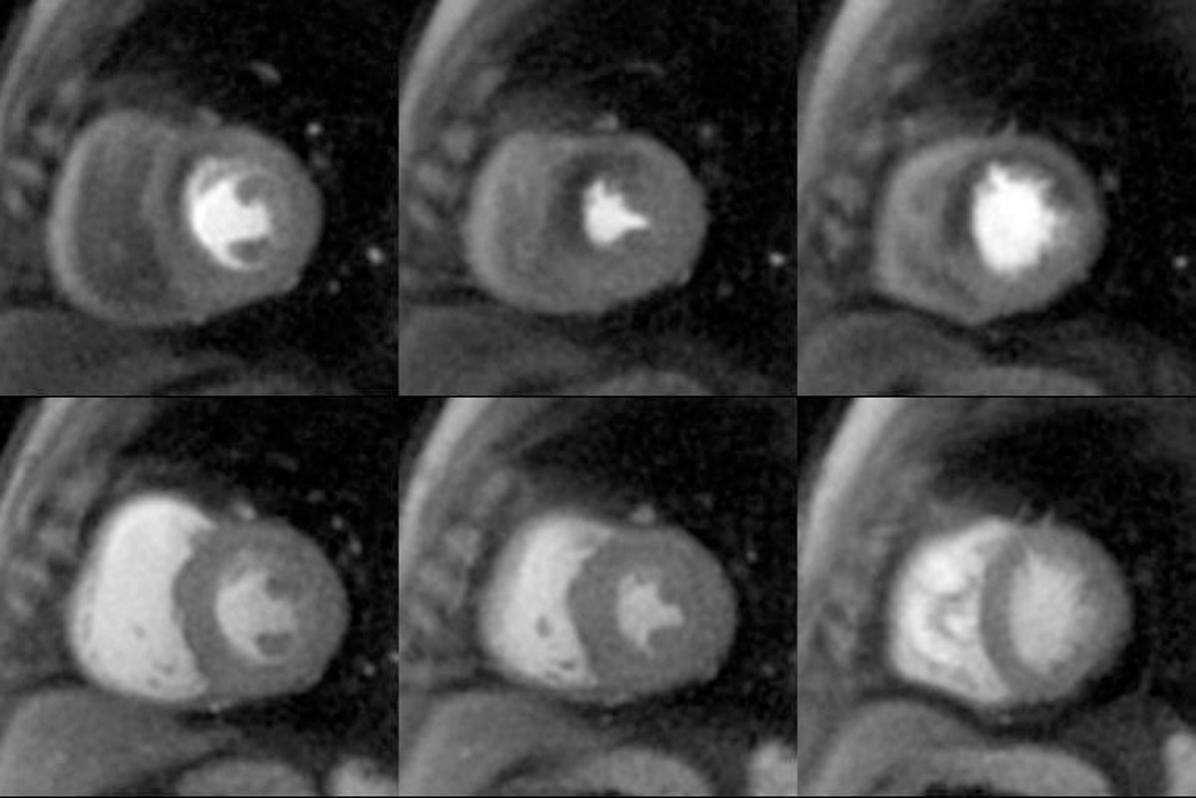
Stress (top) and rest (bottom) perfusion images demonstrate a reversible perfusion defect in the anterior wall and anteroseptum.

Figure 1 shows stress and rest spiral perfusion images from a subject who had normal cardiac function and no LGE on his CMR study. These high quality images show a reversible perfusion abnormality in the anterior wall and anteroseptum. This patient had a 90% stenosis in his LAD at cardiac catheterization. Overall the prevalence of obstructive CAD was 65% and LGE was present in 35% of the patients. The average image quality score was 4.2±0.8 with no studies showing more than minimal dark rim artifact. The average sensitivity, specificity, and overall accuracy of the two readers were 87%, 94%, and 89% respectively. There was very good inter-reader reliability with a kappa statistic of 0.73.

## Conclusions

Spiral adenosine stress CMR results in high diagnostic accuracy for the detection of obstructive coronary artery disease with excellent image quality.

## Funding

This work was funded by AHA 10SDG2650038 Salerno(PI) and Siemens Medical Solutions.
